# Tomato SR/CAMTA transcription factors SlSR1 and SlSR3L negatively regulate disease resistance response and SlSR1L positively modulates drought stress tolerance

**DOI:** 10.1186/s12870-014-0286-3

**Published:** 2014-10-28

**Authors:** Xiaohui Li, Lei Huang, Yafen Zhang, Zhigang Ouyang, Yongbo Hong, Huijuan Zhang, Dayong Li, Fengming Song

**Affiliations:** National Key Laboratory for Rice Biology, Institute of Biotechnology, Zhejiang University, Hangzhou, 310058 China

**Keywords:** Tomato (*Solanum lycopersicum*), SR/CAMTA, Disease resistance response, Drought stress

## Abstract

**Background:**

The SR/CAMTA proteins represent a small family of transcription activators that play important roles in plant responses to biotic and abiotic stresses. Seven *SlSR*/*CAMTA* genes were identified in tomato as tomato counterparts of SR/CAMTA; however, the involvement of *SlSRs*/*CAMTAs* in biotic and abiotic stress responses is not clear. In this study, we performed functional analysis of the *SlSR*/*CAMTA* family for their possible functions in defense response against pathogens and tolerance to drought stress.

**Results:**

Expression of *SlSRs* was induced with distinct patterns by *Botrytis cinerea* and *Pseudomonas syringae* pv. *tomato* (*Pst*) DC3000. Virus-induced gene silencing (VIGS)-based knockdown of either *SlSR1* or *SlSR3L* in tomato resulted in enhanced resistance to *B. cinerea* and *Pst* DC3000 and led to constitutive accumulation of H_2_O_2_, elevated expression of defense genes, marker genes for pathogen-associated molecular pattern-triggered immunity, and regulatory genes involved in the salicylic acid- and ethylene-mediated signaling pathways. Furthermore, the expression of *SlSR1L* and *SlSR2L* in detached leaves and whole plants was significantly induced by drought stress. Silencing of *SlSR1L* led to decreased drought stress tolerance, accelerated water loss in leaves, reduced root biomass and attenuated expression of drought stress responsive genes in tomato. The SlSR1 and SlSR3L proteins were localized in the nucleus of plant cells when transiently expressed in *Nicotiana benthamiana* and had transcriptional activation activity in yeast.

**Conclusions:**

VIGS-based functional analyses demonstrate that both SlSR1 and SlSR3L in the tomato *SlSR*/*CAMTA* family are negative regulators of defense response against *B. cinerea* and *Pst* DC3000 while SlSR1L is a positive regulator of drought stress tolerance in tomato.

**Electronic supplementary material:**

The online version of this article (doi:10.1186/s12870-014-0286-3) contains supplementary material, which is available to authorized users.

## Background

Plants are vulnerable to various biotic and abiotic stresses but have evolved to equip with sophisticated signaling networks to precisely regulate defense response to unfavorable stresses. Upon perception of environmental stress, a set of early signaling events including changes in the cytosolic free calcium (Ca^2+^) concentration signatures (i.e. oscillations varying in cellular location, amplitude, duration or frequency) is often activated and integrated into different signaling pathways, which ultimately initiate transcriptional reprogramming leading to expression of a large set of stress-responsive genes [1-3]. Extensive biochemical and genetic studies have demonstrated that cellular Ca^2+^ as a universal second messenger plays critical roles in regulating defense responses to diverse biotic and abiotic stresses [4-7].

Cellular Ca^2+^ changes can be sensed and interpreted by calcium-binding proteins (CaBPs) including calmodulin (CaM), calcineurin B-like proteins and calcium-dependent protein kinases [8-10]. These CaBPs regulate cellular responses through two distinct pathways upon sensing different biotic and abiotic signals [8-10]. Firstly, CaBPs trigger rapid responses by direct binding to cytosolic target proteins and modulating their activity. Alternatively, CaBPs modulate indirect and relatively slow cellular responses by interacting with transcription factors to regulate gene expression. Recently, a class of CaM-binding transcription factors (CAMTA for CaM-binding transcription activator) has been identified in plants [11-14]. The CAMTA proteins, also called signal-responsive (SR) proteins, are present in all plant and animal species examined to date and are highly conserved in their protein structures. Typically, the SR/CAMTA proteins contain a CG-1 DNA-binding domain (binding to specific *cis*-elements in promoter regions of the target genes) at the N-terminus, a TIG domain (an immunoglobulin-like fold involved in nonspecific DNA binding), three ankyrin repeats (implicated in protein-protein interaction) and five putative CaM-binding motifs called as IQ motif [11-13]. Biochemical studies with the Arabidopsis AtSR1 and rice OsCBT proteins have identified the primary target of DNA *cis*-element for SR/CAMTA proteins as CGCG and CGTG motifs in promoter regions of the target genes [12,14].

The SR/CAMTA proteins represent a small family of transcription activators in plants. For instance, six genes encoding for SR/CAMTA proteins were identified in Arabidopsis [11]. The *SR*/*CAMTA* genes were shown to be responsive to multiple abiotic and biotic stresses including cold, wounding, drought and pathogen attack, as well as to stress-related hormones like ethylene, auxin, methyl jasmonate (MeJA) and salicylic acid (SA) [11,12,15-18]. Recent genetic studies with loss-of-function and gain-of-function mutants have shown that members of the SR/CAMTA family play important roles in plant response to abiotic and biotic stresses. The Arabidopsis *AtSR1* knockout mutant showed enhanced disease resistance against multiple pathogens with different infection styles including *Pseudomonas syringae* pv. *tomato* (*Pst*), *Botrytis cinerea* and *Golovinomyces cichoracearum* [19-21] but decreased resistance against insect herbivores [22,23]. By contrast, overexpression of *AtSR1* conferred an increased susceptibility to *Pst* DC3000, *B. cinerea* and *G. cichoracearum* [21,24]. Similarly, the rice *oscbt* mutant exhibited significant resistance to blast fungal pathogen *Magnaporthe grisea* and leaf blight bacterial pathogen *Xanthomonas oryzae* pv. *oryzae* [17]. On the other hand, it was recently shown that the Arabidopsis AtSR1 and AtSR2 also play important roles in regulating tolerance to low temperature [25,26] and drought stress [27]. The function of SR/CAMTA proteins in plant biotic and abiotic stress response is achieved mainly through regulating expression of genes whose promoter regions contain the CGCG boxes [20,21,25,27].

Seven *SlSRs/CAMTAs* (hereafter referred to as *SlSRs* for convenience) genes were identified in tomato and were shown to be developmentally regulated during fruit development and ripening and induced by ethylene [28,29]. Further detailed analysis revealed that the *SlSR* genes showed differential expression patterns in tomato fruit in response to low temperature, mechanical injury, infection of the necrotrophic fungal pathogen *B. cinerea*, and treatments with the signaling molecules SA and MeJA [30]. However, direct genetic evidence supporting the involvement of the tomato *SlSRs* in biotic and abiotic stress responses is still lacking. In the present study, we investigated the possible functions of *SlSRs* in disease resistance and drought stress tolerance using virus-induced gene silencing (VIGS) approach. Our VIGS-based functional analyses demonstrate that both SlSR1 and SlSR3L are negative regulators of defense response against *B. cinerea* and *Pst* DC3000 while SlSR1L is a positive regulator of drought stress response.

## Results

### Expression patterns of *SlSRs* in response to pathogen infection

**Figure 1:**
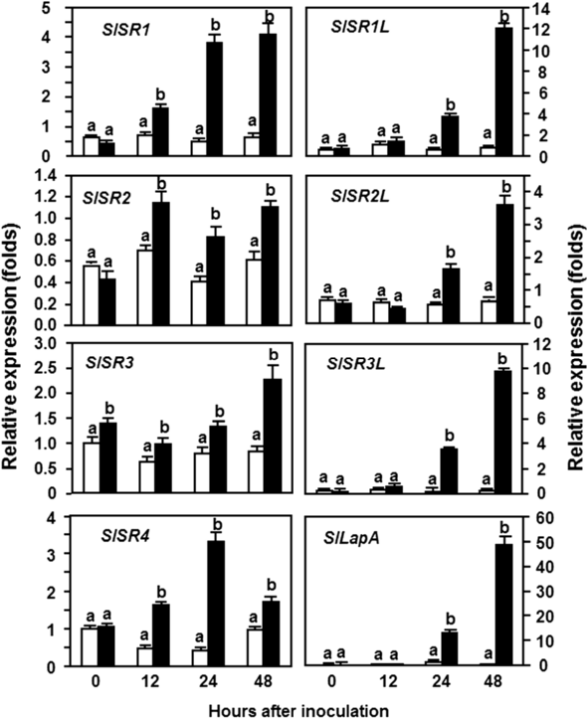
**Expression of** ***SlSRs*** **in response to infection by** ***B. cinerea*** **.** Four-week-old plants were inoculated by foliar spraying with spore suspension (2 × 10^5^ spores/ml) of *B. cinerea* (filled bars) or with same volume of buffer as a mock inoculation control (open bars). Leaf samples were collected at indicated time points after inoculation for analysis of *SlSR* expression by qRT-PCR using gene-specific primers. Relative expression levels were calculated after normalization with actin transcript values. Data presented are the means ± SD from three independent experiments and different letters above the columns indicate significant differences at *p* < 0.05 level between the pathogen-inoculated and mock-inoculated plants.

To explore the possible functions of *SlSRs* in defense response against pathogen infection, we analyzed the expression patterns of *SlSR* genes in tomato plants after infection with different pathogens, *B. cinerea*, a necrotrophic fungal pathogen causing grey mold disease, and *Pst* DC3000, a (hemi) biotrophic bacterial pathogen causing bacterial leaf spot disease. In analysis of expression patterns of *SlSRs* in response to infection of *B. cinerea*, leaf samples collected from the whole plant inoculation assays were used and the expression pattern of *SlLapA*, a defense gene regulated by the JA/ET-mediated signaling pathway [31], was monitored to confirm the efficiency of the inoculation procedure. As shown in Figure 1, the expression level of *SlLapA* in *B. cinerea*-inoculated plants increased significantly, leading to 50 folds of increase at 24 hr post inoculation (hpi) and >300 folds of increase at 48 hpi relative to those in the mock-inoculated plants, suggesting that our inoculation assays were appropriate for further analysis of the expression patterns of *SlSRs* in response to *B. cinerea*. Data from repeated qRT-PCR analyses revealed that the expression of almost all *SlSRs* was induced by infection of *B. cinerea* and the induced expression of *SlSRs* was evident after 24 hpi and showed distinct patterns (Figure 1). The expression levels of *SlSR1*, *SlSR1L*, *SlSR2L* and *SlSR3L* were induced significantly by *B. cinerea*, leading to increases of 8, 8, 3 and 15 folds at 24 hpi and of 8, 20, 7 and 90 folds at 48 hpi, respectively, over those in the mock-inoculated plants (Figure 1). The expression level of *SlSR4* in the *B. cinerea*-inoculated plants showed 3-fold increase at 12 hpi, peaked with 7-fold of increase at 24 hpi and then declined to the level in the mock-inoculated plants (Figure 1). By contrast, the expression levels of *SlSR2* and *SlSR3* in the *B. cinerea*-inoculated plants exhibited slight increases with less than 2 folds as compared with those in the mock-inoculated plants (Figure 1). These results indicate that the expression of *SlSRs* could be induced with distinct patterns by *B. cinerea* and that the expression of most *SlSR* genes except for *SlSR2* and *SlSR3* was highly responsive to infection of *B. cinerea*.

**Figure 2:**
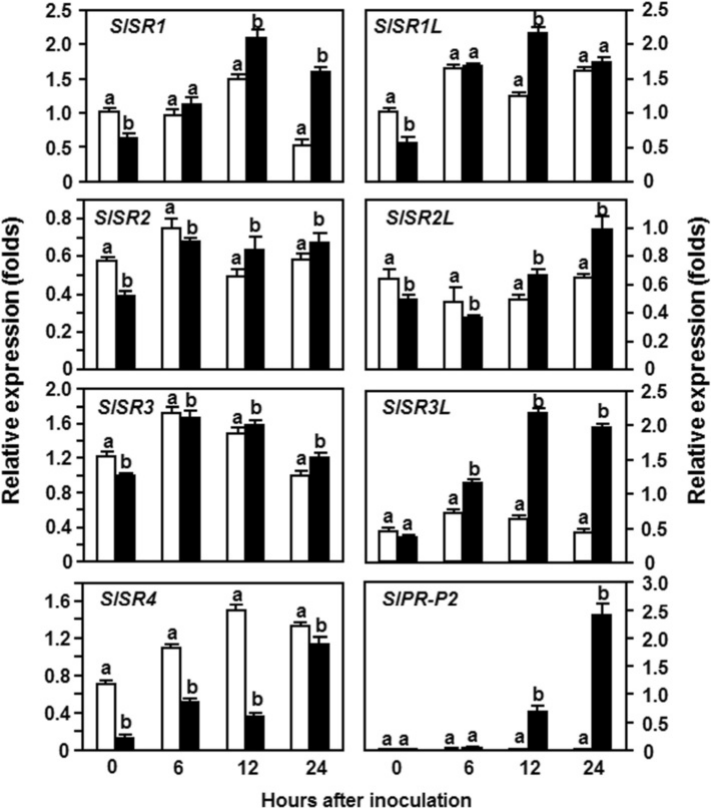
**Expression of** ***SlSRs*** **in response to infection by** ***P. syringae*** **pv.** ***tomato*** **DC3000.** Four-week-old plants were inoculated by vacuum infiltration with *P. syringae* pv. *tomato* DC3000 (OD_600_ = 0.0002) (filled black bars) or with 10 mM MgCl_2_ solution as a mock inoculation control (open bars). Leaf samples were collected at indicated time points after inoculation for analysis of *SlSR* expression by qRT-PCR using gene-specific primers. Relative expression folds were calculated after normalization with actin transcript values. Data presented are the means ± SD from three independent experiments and different letters above the columns indicate significant differences at *p* < 0.05 level the pathogen-inoculated and mock-inoculated plants.

We next analyzed the expression patterns of *SlSRs* in response to a virulent strain of *Pst* DC3000. In these experiments, the expression pattern of *SlPR-P2*, a defense gene regulated by the SA-mediated signaling pathway [31], was examined to confirm the efficiency of the inoculation procedure. As shown in Figure 2, the expression level of *SlPR-P2* in the *Pst* DC3000-inoculated plants increased at 6 hpi and showed 50 and >300 folds of increase at 12 and 24 hpi, over those in the mock-inoculated plants, confirming that the effectiveness of the inoculation procedure was satisfied for further analysis of the expression patterns of *SlSRs* in response to *Pst* DC3000. The expression levels of *SlSR1L*, *SlSR2*, *SlSR2L* and *SlSR3* in the *Pst* DC3000-inoculated plants were similar to those in the mock-inoculated plants, indicating that their expression was not responsive to infection of *Pst* DC3000 (Figure 2). However, the expression levels of *SlSR1* and *SlSR3L* were significantly induced by *Pst* DC3000 (Figure 2). A 3-fold increase of the expression of *SlSR1* in the *Pst* DC3000-inoculated plants was observed at 24 hpi but no significant increase in the expression level of *SlSR1* in the *Pst* DC3000-inoculated plants was observed within the first 12 hpi, as compared with those in the mock-inoculated plants (Figure 2). The expression level of *SlSR3L* in the *Pst* DC3000-inoculated plants exhibited 2-fold and 4-fold increases at 12 hpi and 24 hpi, respectively, relative to those in the mock-inoculated plants (Figure 2). Interestingly, the expression of *SlSR4* in the *Pst* DC3000-inoculated plants was suppressed by *Pst* DC3000 during the first 12 hpi and this suppression of *SlSR4* expression was very quick as the expression level of *SlSR4* in the *Pst* DC3000-inoculated plants decreased by approximately 4 folds relative to that in the mock-inoculated plants (Figure 2). The expression level of *SlSR4* in the *Pst* DC3000-inoculated plants restored to the level in the mock-inoculated plants (Figure 2). These results indicate that the expression of *SlSRs* could be induced with distinct patterns by *Pst* DC3000 and that the expression of *SlSR1* and *SlSR3L* was induced but the expression of *SlSR4* was suppressed by *Pst* DC3000.

### Silencing of *SlSR1* and *SlSR3L* conferred an increased resistance to *B. cinerea* and *Pst* DC3000

**Figure 3:**
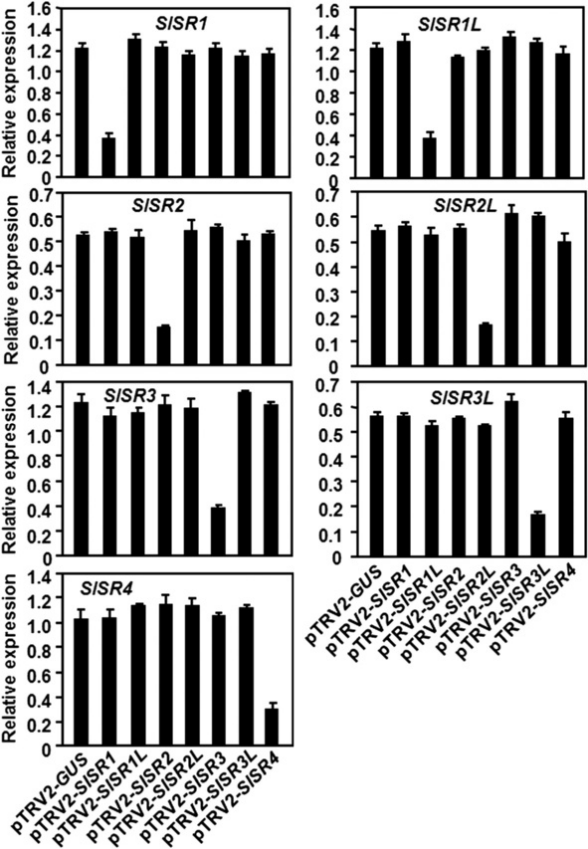
**Silencing efficiency and specificity for target genes in silenced plants.** Two-week-old tomato seedlings were infiltrated with agrobacteria carrying pTRV2-SlSRs or pTRV2-GUS constructs and leaf samples were collected from pTRV2-SlSRs- and pTRV2-GUS-infitlrated plants at 4 weeks after agroinfiltration. Expression levels of each *SlSR* genes in targeted and nontargeted *SlSR* genes-silenced and non-silenced plants were analyzed by qRT-PCR and data obtained were normalized with actin transcript values. Data presented are the means ± SD from three independent experiments.

To explore the possible functions of *SlSRs* in plant defense response, we used the TRV-based VIGS system [32] to knockdown the expression levels of *SlSR* genes in tomato plants and compared the phenotypes and severity of diseases caused by *B. cinerea* and *Pst* DC3000, respectively. In our VIGS study, we chose sequences that encode for the highly diverged regions in the SlSR proteins to avoid interference with expression of other non-target *SlSR* genes when one target *SlSR* gene was attempted to be silenced (Additional file 1). Silencing efficiency and specificity were assessed by qRT-PCR analyzing the transcript abundance of the target *SlSR* gene in the silenced and non-silenced pTRV2-GUS-infiltrated control plants. As shown in Figure 3, the transcript levels of each *SlSR* gene in the corresponding silenced plants were significantly reduced, leading to the silencing efficiency of 70–75% in standard VIGS experiments, as compared with those in the pTRV2-GUS-infiltrated plants. By contrast, the transcript levels of each *SlSR* gene in those plants that were silenced for one of the other six *SlSR* genes were comparable to the level in the pTRV2-GUS-infiltrated control plants (Figure 3), indicating that silencing only occurred for the targeted gene but not for the other non-targeted *SISR* genes. Thus, the silencing efficiency and specificity under our experiment conditions were satisfied for further study and all the subsequent experiments were performed only on those pTRV2-SlSR-infiltrated plants with high levels of silencing efficiency (>70%).

**Figure 4:**
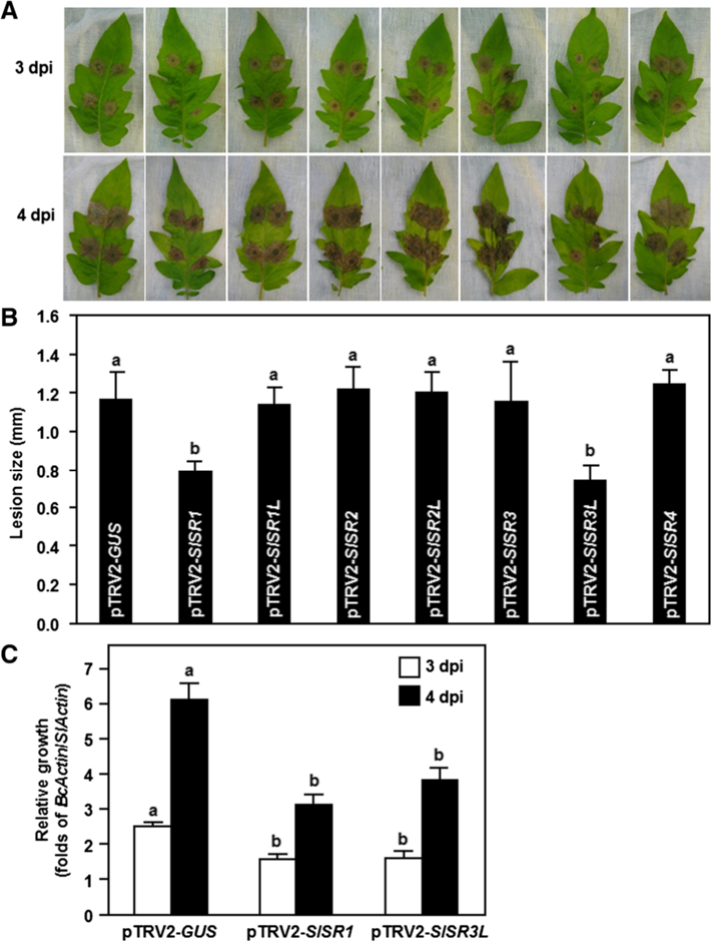
**Silencing of either** ***SlSR1*** **or** ***SlSR3L*** **resulted in enhanced disease resistance to** ***B. cinerea*** **.** Two-week-old tomato seedlings were infiltrated with agrobacteria carrying pTRV2-SlSRs or pTRV2-GUS constructs and leaves were detached from pTRV2-GUS- or pTRV2-SlSR-infiltrated plants at 3 weeks after VIGS infiltration. Inoculation with *B. cinerea* was done by dropping 5 μl of spore suspension (1 × 10^5^ spores/ml). **(A)** Disease symptom on detached leaves at 3 days after inoculation. **(B)** Lesion size in leaves of the pTRV2-GUS- or pTRV2-SlSR-infiltrated plants at 4 days after inoculation. At least 10 leaves from ten individual plants were used for each experiment. **(C)** Growth of *B. cinerea* in inoculated plants from whole plant inoculation experiments. Fungal growth *in planta* was assumed at 3 and 4 days after inoculation by qRT-PCR analyzing the transcript level of *B. cinerea BcActinA* gene using *SlActin* gene as an internal control. Relative fungal growth was shown as folds of transcript levels of *BcActin* compared to *SlActin*. Data presented are the means ± SD from three independent experiments and different letters above the columns indicate significant differences at *p* < 0.05 level.

We first examined the possible roles of *SlSRs* in resistance against *B. cinerea* by challenging the pTRV2-SlSRs-infiltrated plants with spore suspension of *B. cinerea* and comparing the disease severity and *in planta* fungal growth with those in pTRV-GUS-infiltrated non-silenced plants. In our detached leaf assays, *B. cinerea*-caused lesions on detached leaves from the pTRV2-SlSR1L-, pTRV2-SlSR2-, pTRV2-SlSR2L-, pTRV2-SlSR3- and pTRV2-SlSR4-infiltrated plants were similar to the lesions on the detached leaves from pTRV2-GUS-infilrtratd plants (Figure 4A and B), suggesting that *SlSR1L*, *SlSR2*, *SlSR2L*, *SlSR3* and *SlSR4* may not be involved in disease resistance against *B. cinerea*. However, *B. cinerea*-caused lesions on detached leaves from the pTRV2-SlSR1- and pTRV2-SlSR3L-infiltrated plans developed slowly and were still separated, as compared with the large merged lesions on leaves from the pTRV2-GUS-infiltrated plants, at 4 days after inoculation (dpi) (Figure 4A). At 4 dpi, the lesion sizes on detached leaves from the pTRV2-SlSR1- and pTRV2-SlSR3L-infiltrated plants were significantly reduced, leading to a reduction of approximately 35%, as compared with that of the pTRV2-GUS-infiltrated plants (Figure 4B). We further analyzed and compared the *in planta* fungal growth in the pTRV2-SlSR1-, pTRV2-SlSR3L- and pTRV2-GUS-infiltrated plants after inoculation by foliar spraying with spore suspension of *B. cinerea* in whole plant inoculation experiments. qRT-PCR analysis of the transcript levels of the *B. cinerea* actin gene *BcActin*, which was used as an indicative of the rate of fungal growth *in planta*, showed that the fungal growth in the pTRV2-SlSR1- and pTRV2-SlSR3L-infilrated plants were significantly suppressed, resulting in reductions of 35–51% at 3 and 4 dpi, as compared with those in the pTRV2-GUS-infiltrated plants (Figure 4C). These results indicate that the *SlSR1*- and *SlSR3L*-silenced plants were more resistant to *B. cinerea* infection than the pTRV2-GUS-infiltrated plants. Taken together, these data demonstrate that silencing of *SlSR1* or *SlSR3L* resulted in increased resistance against *B. cinerea* and thus both SlSR1 and SlSR3L may act as negative regulators of disease resistance against *B. cinerea*.

**Figure 5:**
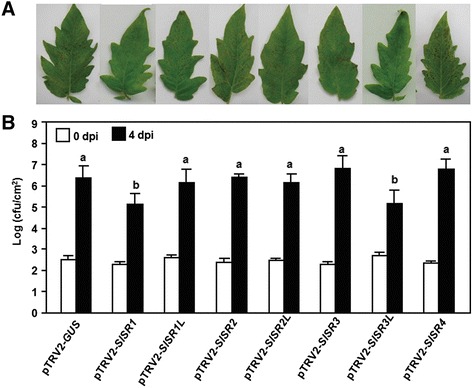
**Silencing of either** ***SlSR1*** **or** ***SlSR3L*** **led to enhanced resistance to** ***P. syringae*** **pv.** ***tomato*** **DC3000.** Two-week-old tomato seedlings were infiltrated with agrobacteria carrying pTRV2-SlSRs or pTRV2-GUS constructs and the pTRV2-GUS- and pTRV2-SlSRs-infiltrated plants were inoculated by vacuum infiltration with infiltration with *P. syringae* pv. *tomato* DC3000 (OD_600_ = 0.0002) at 3 weeks after VIGS infiltration. **(A)** Representative symptom of disease caused by *P. syringae* pv. *tomato* DC3000 at 4 days after inoculation. **(B)** Bacterial growth in inoculated leaves of pTRV2-GUS- and pTRV2-SlSRs-infiltrated plants. Leaf samples were collected at 0 and 4 days after inoculation and bacterial growth was measured. Data presented are the means ± SD from three independent experiments and different letters above the columns indicate significant differences at *p* < 0.05 level.

We next explored whether *SlSRs* have functions in resistance against *Pst* DC3000. Disease phenotypes and bacterial growth *in planta* were compared between the pTRV2-SlSRs- and pTRV2-GUS-infiltrated plants after inoculation with a virulent strain of *Pst* DC3000. At 4 dpi, the pTRV2-GUS-infiltrated plants showed typical bacterial speck disease symptoms, including necrotic lesions surrounded by chlorosis (Figure 5A). The disease severity and bacterial growth *in planta* in inoculated pTRV2-SlSR1L-, pTRV2-SlSR2-, pTRV2-SlSR2L-, pTRV2-SlSR3- and pTRV2-SlSR4-infiltrated plants were comparable to those in the pTRV2-GUS-inflitrated plants (Figure 5A and B), suggesting that *SlSR1L*, *SlSR2*, *SlSR2L*, *SlSR3* and *SlSR4* are not involved in disease resistance to *Pst* DC3000. By contrast, the pTRV2-SlSR1- and pTRV2-SlSR3L-infiltrated plants showed very weak visible symptoms of disease caused by *Pst* DC3000, as compared with that in the pTRV2-GUS-infiltrated plants (Figure 5A). This reduced disease symptoms on leaves of the pTRV2-SlSR1- and pTRV2-SlSR3L-infiltrated plants were coincided with relatively low levels of bacterial growth *in planta* after inoculation with *Pst* DC3000. At 4 dpi, the bacterial populations in leaves of the pTRV2-SlSR1- and pTRV2-SlSR3L-infiltrated plants were 2.8 × 10^5^ and 2.2 × 10^5^ CFU/cm^2^, leading to a reduction of 10-fold, as compared to that in leaves of the pTRV-GUS-infiltrated plants (2.5 × 10^6^ CFU/cm^2^) (Figure 5B). These results demonstrate that silencing of either *SlSR1* or *SlSR3L* led to increased resistance against *Pst* DC3000 and thus both of SlSR1 and SlSR3L are also negative regulators of disease resistance to *Pst* DC3000.

### Silencing of *SlSR1* and *SlSR3L* resulted in constitutive defense response

**Figure 6:**
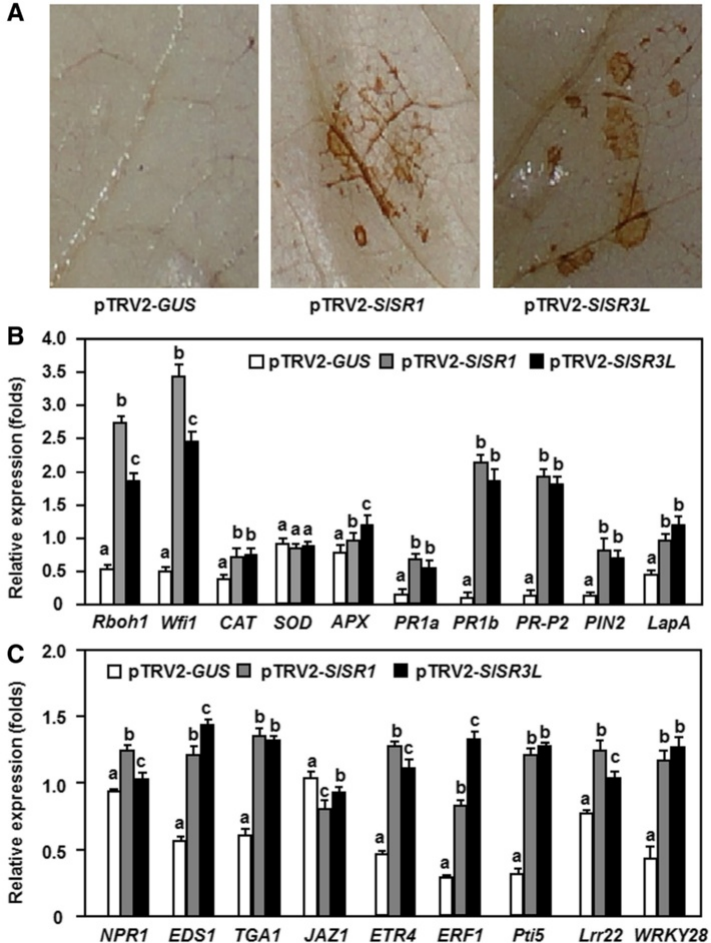
**Silencing of either** ***SlSR1*** **or** ***SlSR3L*** **activated constitutive defense response.** Two-week-old seedlings were infiltrated with agrobacteria carrying pTRV2-SlSR1, pTRV2-SlSR3L or pTRV2-GUS constructs and leaf samples were collected at 2 weeks after VIGS infiltration for detection of H_2_O_2_ accumulation and analysis of expression of signaling- and defense-related genes. **(A)** Accumulation of H_2_O_2_ in leaves of the pTRV2-SlSR1- and pTRV2-SlSR3L-infiltrated plants. **(B)** Expression of defense-related genes and ROS production-related genes in the pTRV2-SlSR1- and pTRV2-SlSR3L-infiltrated plants. **(C)** Expression of genes involved in different defense signaling pathways in the pTRV2-SlSR1- and pTRV2-SlSR3L-infiltrated plants. Data presented are the means ± SD from three independent experiments and different letters above the columns indicate significant differences at *p* < 0.05 level.

The Arabidopsis *sr1* mutant plants were previously found to display chlorosis and constitutive expression of defense genes under lower temperature, indicating that loss of SR1 function in Arabidopsis led to constitutive defense response [20]. To examine whether silencing of *SlSR1* or *SlSR3L* could also confer constitutive defense responses, we analyzed and compared the accumulation of H_2_O_2_ and expression of genes involved in different signaling pathways and defense response between the pTRV2-SlSR1- and pTRV2-SlSR3L-infiltrated plants and the pTRV2-GUS-infiltrated plants. Results from 3,3-diaminobenzidine (DAB) staining of *in situ* H_2_O_2_ accumulation showed that significant brown precipitates, representing the accumulation of H_2_O_2_, were easily and clearly observed in leaves of the pTRV2-SlSR1- and pTRV2-SlSR3L-infiltrated plants without infection of pathogen, while no significant brown precipitate was seen in leaves of the pTRV2-GUS-infiltrated plants (Figure 6A). These results indicate that silencing of either *SlSR1* or *SlSR3L* resulted in constitutive accumulation of H_2_O_2_ in tomato plants. To explore the possible mechanism for the constitutive accumulation of H_2_O_2_ in the *SlSR1*- and *SlSR3L*-silencedd plants, we analyzed and compared the expression of genes encoding for NADPH oxidases, which are plasma membrane-localized ROS generating enzymes [33], and for catalases (CAT), superoxide dismutases (SOD) and ascorbate peroxidases (APX), which are involved in scavenging of ROS, in the pTRV2-SlSR1- and pTRV2-SlSR3L-infiltrated plants. As shown in Figure 6B, the expression levels of *Rboh1* and *Wfi1*, two genes for NADPH oxidases, in the pTRV2-SlSR1- and pTRV2-SlSR3L-infiltrated plants were significantly elevated, giving increases of 5 ~ 7-fold for *Rboh1* and 4 ~ 5-fold for *Wfi1*, as compared with those in the pTRV2-GUS-infiltrated plants. Similarly, the expression levels of *CAT* and *APX* in the pTRV2-SlSR1- and pTRV2-SlSR3L-infiltrated plants were also increased as compared with those in the pTRV2-GUS-infiltrated plants (Figure 6B). By contrast, no significant difference was observed in the expression level of *SOD* between the pTRV2-SlSR1- and pTRV2-SlSR3L-infiltrated plants and the pTRV2-GUS-infiltrated plants (Figure 6B). These results indicate that the constitutive accumulation of H_2_O_2_ in the *SlSR1*- and *SlSR3L*-silenced plants might be attributed to the increased ROS generating ability resulted from the high level of expression of the NADPH oxidases.

We next examined whether silencing of *SlSR1* or *SlSR3L* led to constitutive expression of defense genes. The expression levels of five representative defense genes that are regulated through different defense signaling pathways and three marker genes for pathogen-associated molecular patterns-triggered immunity (PTI) were analyzed and compared between the pTRV2-SlSR1- and pTRV2-SlSR3L-infiltrated plants and the pTRV2-GUS-infiltrated plants. As shown in Figure 6B, the expression levels of *PR1a*, *PR1b* and *PR-P2*, which are thought to be regulated by the SA-mediated signaling pathway [31], and of *PIN2* and *LapA*, which are considered to be regulated by the JA/ET signaling pathway [31], were increased significantly in the pTRV2-SlSR1- and pTRV2-SlSR3L-infiltrated plants, leading to 8 ~ 25 folds of increases for the *PR1a*, *PR1b* and *PR-P2* genes and 1 ~ 11 folds of increases for the *PIN2* and *LapA* genes, as compared with those in the pTRV2-GUS-infiltrated plants. Similarly, the expression levels of *Pti5*, *Lrr22* and *WRKY28*, three PTI marker genes in tomato [34,35], in the pTRV2-SlSR1- and pTRV2-SlSR3L-infiltrated plants were significantly higher than those in the pTRV2-GUS-infiltrated plants, resulting in increases of 0.5 ~ 3.0 folds in the expression levels (Figure 6C). These data demonstrate that silencing of either *SlSR1* or *SlSR3L* activates constitutively the expression of the defense and PTI genes, leading to constitutive defense response in the *SlSR1*- and *SlSR3L*-silenced plants.

To determine whether silencing of *SlSR1* and *SlSR3L* activates defense signaling pathways, we further analyzed and compared the expression levels of the key genes encoding important components involved in the SA- and JA/ET-mediated signaling pathways between the pTRV2-SlSR1- and pTRV2-SlSR3L-infiltrated plants and the pTRV2-GUS-infiltrated plants. The expression levels of *NPR1*, *EDS1* and *TGA1*, known to be critical components in the SA-mediated signaling pathway [36], in the pTRV2-SlSR1- and pTRV2-SlSR3L-infiltrated plants were significantly increased, especially for the expression levels of the *EDS1* and *TGA1* genes, showing 1-fold increase relative to those in the pTRV2-GUS-infiltrated plants (Figure 6C). Similarly, the expression levels of *ETR4* and *ERF1*, known to be associated with ET signaling pathway [37,38], in the pTRV2-SlSR1- and pTRV2-SlSR3L-infiltrated plants showed 1 ~ 2-fold increase over those in the pTRV2-GUS-infiltrated plants (Figure 6C). By contrast, the expression level of *JAZ1*, known to be associated with JA signaling pathway [39], in the pTRV2-SlSR1- and pTRV-SlSR3L-infiltrated plants was reduced as compared with those in the pTRV2-GUS-infiltrated plants (Figure 6C). These results suggest that silencing of either *SlSR1* or *SlSR3L* can activate both the SA- and ET-mediated signaling pathways but suppress the JA-mediated signaling pathway in the *SlSR1*- and *SlSR3L*-silenced plants.

### Expression patterns of *SlSRs* in response to drought stress and ABA

**Figure 7:**
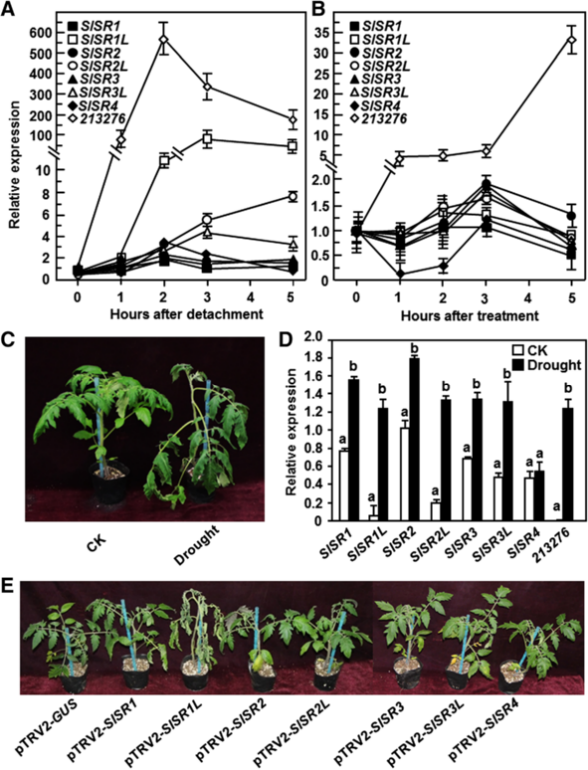
**Expression patterns of** ***SlSRs*** **in response to drought stress and ABA treatment and phenotypes of the** ***SlSRs*** **-silenced plants under drought condition. (A)** Expression of *SlSRs* in detached leaves under drought stress. Fully expanded leaves were detached from four-week-old plants and subjected to drought stress treatment by placing on lab blench or water-saturated filter papers in Petri dishes as a control and samples were collected at different time points as indicated. **(B)** Expression of *SlSRs* in detached leaves after ABA treatment. Four-week-old plants were treated by foliar spraying with ABA solution (100 μM) or water as control and leaf samples were collected at different time points as indicated. Relative expression levels of the *SlSR* genes in the treated plants were shown as folds of the expression levels in the control plants after normalization with actin transcript values. **(C)** and **(D)** Expression of *SlSRs* in leaves of plants under drought stress. Four-week-old plants were treated for drought stress by stopping watering for a period or watered normally as controls and leaf samples were collected at 7 days after treatment when wilting symptom appeared **(C)**. Total RNA was extracted and used for qRT-PCR analysis of expression of *SlSRs* **(D)**. Relative expression levels of the *SlSR* genes in the treated and control plants were shown as folds of the actin transcript values. **(E)** Phenotypes of the *SlSRs*-silenced plants under drought condition. Two-week-old seedlings were infiltrated with agrobacteria carrying pTRV2-SlSRs or pTRV2-GUS constructs and 3 weeks later the pTRV2-GUS- and pTRV2-SlSRs-infiltrated plants were subjected to drought stress by stopping watering for 10 days. Data presented in **(A)**, **(B)** and **(D)** are the means ± SD from three independent experiments and different letters above the columns in **(D)** indicate significant difference at *p* < 0.05 level.

To explore the possible involvement of *SlSRs* in drought stress response, we first examined whether the expression of *SlSRs* could be induced by drought stress and abscisci acid (ABA) treatment. Two different methods, the detached leaf and the whole plant assays, were adapted to analyze the expression of *SlSRs* in response to drought stress. In these experiments, a previously reported drought stress-responsive gene, *SGN-213276* [40], was included to confirm the efficiency of the drought stress treatment. The expression level of *SGN-213276* was markedly increased after drought stress treatment, leading to increases of >100-fold at 5 hr after treatment in the detached leaf assays (Figure 7A) and at 7 days after treatment in the whole plant assays (Figure 7D), indicating that the experiments for drought stress assays were satisfied for further analyzing the expression of the *SlSR* genes. In the detached leaf assays, as compared with the expression levels of the corresponding genes in the water-saturated detached leaves, no significant change in the expression levels of *SlSR1*, *SlSR2*, *SlSR3* and *SlSR4* was observed over a period of 5 hr after detachment (Figure 7A), whereas a maximal increase of 4 ~ 8-fold in the expression levels of *SlSR2L* and *SlSR3L* was observed during 3–5 hr after treatment (Figure 7A). By contrast, the expression kinetic of *SlSR1L* under drought stress condition was similar to that of the *SGN-213276* gene (Figure 7A). The expression level of *SlSR1L* in the drought stress treated leaves started to increase at 2 hr after detachment and exhibited approximately an increase of 50 ~ 90 folds as compared with that in the water-saturated leaves (Figure 7A). In the whole plant assays, the drought stressed plants exhibited clear wilting symptom at 7 days after withholding water whereas the normally watered plants did not show any wilting symptom (Figure 7C). qRT-PCR analyses revealed that the expression of *SlSRs* except *SlSR4* was induced by drought stress. The expression levels of *SlSR1*, *SlSR2*, *SlSR3* and *SlSR3L* in the drought stressed plants showed a slight increase, leading to an increase of approximately 1 fold or less over those in the normally watered plants (Figure 7D). However, the expression levels of *SlSR1L* and *SlSR2L* in the drought stressed plants increased significantly as compared with those in the normally watered plants, resulting in increases of 20 folds and 7 folds, respectively (Figure 7D). Taken together, these results suggest that some *SlSRs* especially *SlSR1L* are responsive to drought stress. We also analyzed whether the expression of *SlSRs* could be induced by exogenous application of ABA, a well-known hormone involved in drought stress response [41]. In these experiments, the expression level of the drought stress-responsive gene *SGN-213276* in the ABA-treated plants was markedly increased, leading to increases of 5 ~ 34-fold over those in the control plants (Figure 7B); however, the expression levels of *SlSRs* in the ABA-treated plants showed changes with less 2 folds as compared with those in the control plants (Figure 7B), indicating that exogenous ABA did not affect the expression of *SlSRs*.

### Silencing of *SlSR1L* reduced drought tolerance in tomato

**Figure 8:**
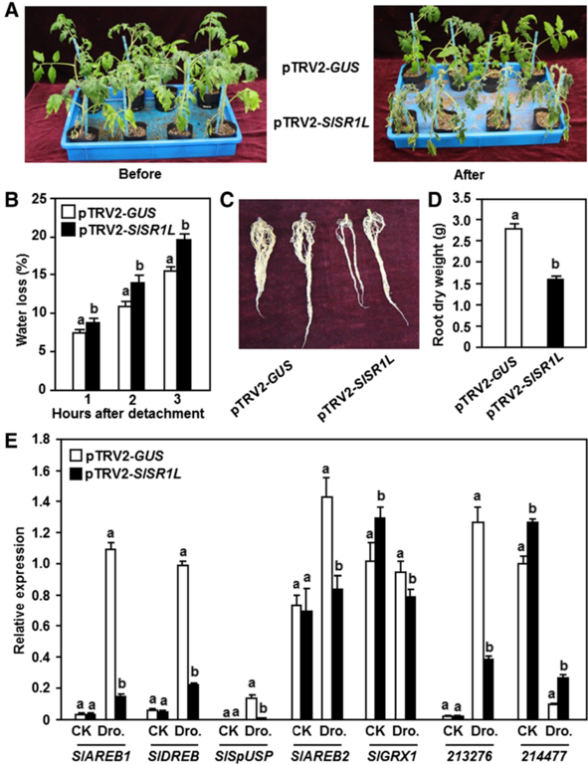
**Silencing of** ***SlSR1L*** **led to reduced drought stress tolerance.** Two-week-old tomato seedlings were infiltrated with agrobacteria carrying pTRV2-SlSR1L or pTRV2-GUS constructs and 3 weeks later the pTRV2-GUS- and pTRV2-SlSR1L-infiltrated plants were subjected to drought stress by stopping watering. **(A)** Phenotype of the pTRV2-GUS- and pTRV2-SlSR1L-infiltrated plants before and after treatment of drought stress. **(B)** Rates of water loss in detached leaves of the pTRV2-GUS- and pTRV2-SlSR1L-infiltrated plants. **(C)** Root system of the pTRV2-GUS- and pTRV2-SlSR1L-infiltrated plants. The intact root systems are shown from one representative pTRV2-GUS- or pTRV2-SlSR1L-infiltrated plant. **(D)** Dry weights of roots from the pTRV2-GUS- and pTRV2-SlSR1L-infiltrated plants. **(E)** Expression of some drought-responsive genes in the pTRV2-GUS- and pTRV2-SlSR1L-infiltrated plants before and after treatment of drought stress. Data presented in **(B)**, **(C)** and **(E)** are the means ± SD from three independent experiments and different letters above the columns indicate significant differences at *p* < 0.05 level.

VIGS assays were performed to explore the involvement of individual *SlSRs* in drought stress response. For this purpose, we stopped watering for 7–10 days to compare the phenotype between the pTRV2-SlSRs- and pTRV2-GUS-inflitrated plants. In repeated experiments, silencing of *SlSR1*, *SlSR2*, *SlSR2L*, *SlSR3*, *SlSR3L* or *SlSR4* did not affect drought stress response over an experimental period of 10 days (Figure 7E), indicating that these *SlSR* genes may not be involved in drought stress response. However, the pTRV-SlSR1L-infiltrated plants showed significant wilting symptom at 7 days after drought stress treatment as compared with that of the pTRV2-GUS-infiltrated plants (Figure 7E), implying a role for *SlSR1L* in drought stress tolerance. The pTRV2-SlSR1L-infiltrated plants grew well as the pTRV2-GUS-infiltrated plants before withholding water, but they were easier to appear wilting symptom after stopping watering and their leaves became curly and the plants wilted at 7 days after withholding water (Figure 8A). This result indicates that silencing of *SlSR1L* attenuated the drought stress tolerance in tomato. To explore the possible mechanism for the reduced drought stress tolerance in the *SlSR1L*-silenced plants, we first analyzed and compared the physiological and morphological changes between the pTRV2-SlSR1L- and pTRV2-GUS-infiltrated plants before and after drought stress treatment. The rate of water loss in leaves from the pTRV2-SlSR1L-infiltrated plants was higher than those in leaves from the pTRV2-GUS-infiltrated plants during the first 3 hr after detachment (Figure 8B), indicating that silencing of *SlSR1L* accelerated water loss in leaves. The pTRV2-SlSR1L-infiltrated plants had smaller root system as compared to the pTRV2-GUS-infiltrated plants (Figure 8C). Similarly, dry weights of the roots from the pTRV2-SlSR1L-infiltrated plants were significantly lower than that of the roots from the pTRV2-GUS-infiltrated plants, resulting in a reduction of approximately 40% (Figure 8D). Furthermore, we also analyzed and compared the expression of some previously reported drought stress-responsive genes *SlAREB1* [42], *SlAREB2* [42], *SlDREB* [43], *SlSpUSP* [44], *SlGRX1* [45], *SGN-213276* [40] and *SGN-214777* [40] in the pTRV2-SlSR1L- and pTRV2-GUS-infiltrated plants before and after drought stress treatment. Before drought stress treatment, the expression levels of *SlAREB1*, *SlAREB2*, *SlDREB*, *SlSpUSP* and *SGN-213276* in the pTRV2-SlSR1L-infiltrated plants were comparable to those in the pTRV2-GUS-infiltrated plants, whereas the expression levels of *SlGRX1* and *SGN-214777* the pTRV2-SlSR1L-infiltrated plants showed a slight increase as compared with those in the pTRV2-GUS-infiltrated plants (Figure 8E). At 10 days after drought stress treatment, the expression levels of *SlAREB1*, *SlDREB*, *SlSpUSP*, *SlAREB2* and *SGN-213276* in the pTRV2-GUS-infiltrated plants increased significantly as compared with those in the normally watered control plants (Figure 8E). After drought stress treatment, however, the expression levels of *SlAREB1*, *SlDREB*, *SlSpUSP*, *SlAREB2* and *SGN-213276* in the pTRV2-SlSR1L-infiltrated plants decreased markedly as compared with those in the pTRV2-GUS-infiltrated plants (Figure 8E). The expression level of *SGN-214777* decreased in the pTRV2-GUS-infiltrated plants as compared with that in the normally watered plants but increased significantly in the pTRV2-SlSR1L-infiltrated plants as compared with that in the pTRV2-GUS-infiltrated plants after drought stress treatment (Figure 8E).

### SlSR1 and SlSR3L are localized in nucleus and have transactivation activity

**Figure 9:**
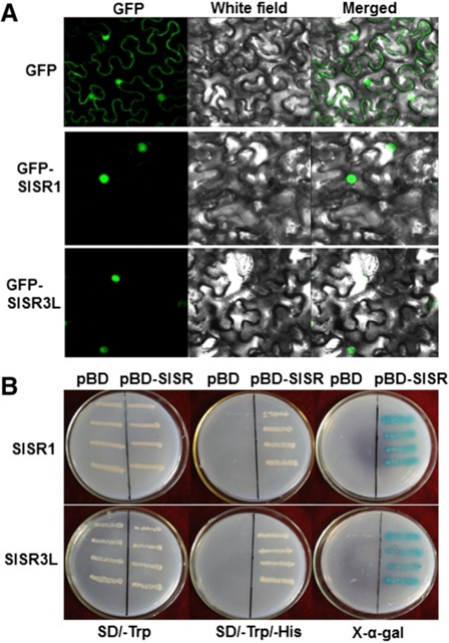
**Subcellular localization and transactivation activity of SlSR1 and SlSR3L proteins. (A)** SlSR1 and SlSR3L are localized in nucleus. Agrobacteria carrying pFGC-Egfp-SlSR1, pFGC-Egfp-SlSR3L or pFGC-Egfp were infiltrated into *N. benthamiana* leaves and the images were taken in dark field for green fluorescence (left), in white field for the morphology of the cell (middle), and in combination (right), respectively. **(B)** SlSR1 and SlSR3L have transactivation activity. Yeast cells carrying pBD-SlSR1, pBD-SlSR3L or pBD empty vector (as a negative control) were streaked on SD/–Trp plates (left) or SD/–Trp/–His plates (middle) for 3 days at 28°C. The x-α-gal was added to the SD/-Trp/-His plates and kept at 28°C for 6 hr (right).

Because of the importance of SlSR1 and SlSR3L in defense response against *B. cinerea* and *Pst* DC3000, we investigated the biochemical characteristics of these two SlSR proteins. Firstly, we examined the subcellular localization of SlSR1 and SlSR3L using a transient expression approach. We transiently expressed SlSR1 and SlSR3L in leaves of 4-week-old *N. benthamiana* plants by infiltration with agrobacteria carrying pFGC-Egfp-SlSR1, pFGC-Egfp-SlSR3L or pFGC-Egfp constructs and GFP was observed at 2 days after agroinfiltration. As shown in Figure 9A, the SlSR1-GFP and SlSR3L-GFP fusions accumulated exclusively in the nucleus of *N. benthamiana* cells, whereas the GFP protein alone accumulated in both the cytoplasm and the nucleus, demonstrating that both SlSR1 and SlSR3L proteins are localized in the nucleus of cells. Furthermore, we also examined whether the SlSR1 and SlSR3L proteins had transactivation activity using a yeast assay system. As shown in Figure 9B, all yeast transformants grew well on SD/–Trp medium. However, only yeast transformants containing pBD-SlSR1 or pBD-SlSR3L were able to grow on the SD/–Trp/–His medium and produced a blue pigment after the addition of x-α-gal, showing a β-galactosidase activity, whereas transformants containing the pBD empty vector did not. These results indicate that both SlSR1 and SlSR3L have transactivation activity in yeasts. Taken together, our experimental data demonstrate that both SlSR1 and SlSR3L are nucleus-localized transcriptional activators.

## Discussion

Previous studies have identified a total of seven *SlSRs* genes in tomato and found that expression of *SlSRs* was regulated in tomato fruit by developmental cues and by biotic and abiotic environmental stress signals [29,30]; however, the biological functions of *SlSRs* in tomato response to biotic and abiotic stress remain unclear. In the present study, data from our VIGS-based functional analyses demonstrate that both SlSR1 and SlSR3L act as negative regulators of defense response against *B. cinerea* and *Pst* DC3000 while SlSR1L functions as a positive regulator of drought stress tolerance in tomato. These findings not only demonstrate that members of the small SlSR family play important roles in regulation of defense responses to biotic and abiotic stresses but also extend our understanding on the biological function of *SlSRs* in regulating stress response.

In plants, members of the SR family have been shown to be induced by infections from different pathogens. For example, the expression of *AtSR1* in Arabidopsis was induced by *Pst* DC3000 and *G. cichoracearum* [21]. In this study, we observed that the expression of *SlSRs* could be induced with different patterns by *Pst* DC3000 and *B. cinerea* (Figures 1 and 2). In response to infection of *B. cinerea*, all the *SlSRs* showed upregulated expression patterns in leaf tissues, especially for *SlSR1*, *SlSR1L*, *SlSR3L* and *SlSR4*, whose expression levels were significantly upregulated (Figure 1). The *B. cinerea*-induced expression patterns of *SlSRs* in leaf tissues are somewhat different from the previous observation that the expressions of *SlSR1*, *SlSR1L*, *SlSR2L* and *SlSR3* in wounded tomato fruit were suppressed by *B. cinerea* [30], probably due to the tissue-specific expression feature of the *SlSRs* [29]. In contrast to the *B. cinerea*-induced expression patterns of *SlSRs*, expression of *SlSR1*, *SlSR1L*, *SlSR3* and *SlSR3L* in leaf tissues was upregulated in response to infection of *Pst* DC3000 (Figure 2). The *Pst* DC3000-induced expression of *SlSR1* is similar to that of the Arabidopsis *AtSR1*, closely related to *SlSR1* [29], whose expression was induced by *Pst* DC3000 [21]. However, infection of *Pst* DC3000 did not affect the expression of *SlSR2* and *SlSR3* but suppressed the expression of *SlSR4* in leaf tissues (Figure 2), which is contrast to the observation that the expression of *SlSR4* in fruit tissues was induced by *B. cinerea* [30]. Therefore, it seems reasonable that the expression of *SlSRs* is precisely controlled by complex mechanisms in response to infection from different pathogens. On the other hand, the *B. cinerea*- and *Pst* DC3000-induced expressions of *SlSRs*, especially for *SlSR1* and *SlSR3L*, were much evident after 24 hr of inoculation (Figures 1 and 2), which is similar to the observation that the expression of *AtSR1* in Arabidopsis leaves was only significantly induced by *Pst* DC3000 after 24 hr of inoculation [21]. It was also found that the expression of most *SlSRs* in tomato fruits was markedly induced by exogenous SA and MeJA only after 8 hr of treatment [30]. These observations suggest that most of the *SlSRs* should belong to late pathogen-responsive genes [30]. The *B. cinerea*- and *Pst* DC3000-induced expression of *SlSR1* and *SlSR3L* in leaves (Figures 1 and 2), along with their SA- and MeJA-induced expression patterns in fruits [30], suggest that SlSR1 and SlSR3L may play important roles in defense response to infection of *B. cinerea* and *Pst* DC3000.

In our VIGS-based functional analyses of *SlSRs* in defense response against different pathogens, we found that silencing of either *SlSR1* or *SlSR3L* resulted in increased disease resistance against *B. cinerea* and *Pst* DC3000, as the *SlSR1*- and *SlSR3L*-silenced plants exhibited less severity of the diseases and supported less *in planta* growth of the pathogens than the non-silenced plants (Figures 4 and 5), indicating that loss-of-function of either *SlSR1* or *SlSR3L* confers a broad-spectrum disease resistance against different pathogens with distinct infection styles. SlSR1 is phylogenetically closely related to the Arabidopsis AtSR1 [29], whose loss-of-function mutant plants showed increased disease resistance against three different pathogens including *Pst* DC3000, *B. cinerea* and *G. cichoracearum* [19-21] and gain-of-function mutant plants exhibited compromised systemic acquired resistance and basal immunity [21,24]. Similar results were also observed in the mutant plants of the *OsCBT* gene, a rice SR family member closely related to *AtSR1* and *SlSR1*, which exhibited significant enhanced resistance to blast fungal pathogen *M. grisea* and leaf blight bacterial pathogen *X. oryzae* pv. *oryzae* [17]. Thus, it is likely that loss-of-function of some of the SR family members can confer broad-spectrum disease resistance in plants. This may have extensive significance that members of the SR family can be used for generating transgenic varieties with broad-spectrum resistance in economically important crops through RNA interference-mediated suppression of expression of target SR genes. Notably, in addition to SR1, the function of other SR family members in disease resistance has not been elucidated so far. In this study, we demonstrated that SlSR3L, like SlSR1 in tomato (Figures 4 and 5) and AtSR1 in Arabidopsis [19-21], acts as a negative regulator of disease resistance against different pathogens in tomato. This finding characterized one member with important function in disease resistance from the relatively small SR family. It will be interesting to examine whether the Arabidopsis homologs of SlSR3L, AtSR3 and AtSR6 [29], play roles in regulating disease resistance. Although expression of *SlSR1L*, *SlSR2*, *SlSR2L* and *SlSR3* was induced by *B. cinerea* and/or *Pst* DC3000 (Figures 1 and 2), silencing of each of these *SlSR* genes had no effect on disease resistance against these two pathogens (Figures 4 and 5). The expression of *SlSR4* was induced by *B. cinerea* but suppressed by *Pst* DC3000 (Figures 1 and 2); however, silencing of *SlSR4* did not lead to any alteration in disease resistance to *B. cinerea* and *Pst* DC3000 (Figures 4 and 5). These results indicate that the *SlSR* genes are highly responsive to pathogen infection but the responsiveness of these *SlSR* genes may be a side effect caused by pathogen infection but not a true reflection for their function in disease resistance.

The observed increased disease resistance against *B. cinerea* and *Pst* DC3000 in the *SlSR1*- and *SlSR3L*-silenced plants may be attributed to an improved basic immunity resulted from loss-of-function of *SlSR1* and *SlSR3L*. This hypothesis is supported by several lines of evidence obtained from some biochemical and molecular analyses toward the *SlSR1*- and *SlSR3L*-silenced plants under pathogen-free conditions. Firstly, the *SlSR1*- and *SlSR3L*-silenced plants constitutively accumulated high level of H_2_O_2_ in leaves, as revealed by *in situ* DAB staining (Figure 6A). Similar phenomenon was also observed in the Arabidopsis *sr1* mutant plants that accumulated high level of H_2_O_2_ in leaves without pathogen infection [19,20]. It was previously found that suppression of *SlWfi1* expression resulted in significant decrease of H_2_O_2_ accumulation in antisense tomato plants [46]. The expression of *SlWfi1* and *SlRboh1*, encoding plasma membrane-localized NADPH oxidases that are involved in generating ROS [33], was significantly upregulated in the *SlSR1*- and *SlSR3L*-silenced plants (Figure 6B), indicating an accelerated generation of ROS in these plants. By contrast, the ROS scavenging capacity in the *SlSR1*- and *SlSR3L*-silenced plants might not be affected significantly as the expression of the *SOD* gene was not changed (Figure 6B). The upregulated expression of the *CAT* and *APX* genes in the *SlSR1*- and *SlSR3L*-silenced plants might be a response to the high level of H_2_O_2_ accumulated in these plants as H_2_O_2_ was found to be capable of mediating the expression of *CAT* genes under stress conditions [47,48]. An accelerated generation with an unchanged scavenging capacity in the *SlSR1*- and *SlSR3L*-silenced plants broke the ROS generating and scavenging balance and favored to accumulate high level of H_2_O_2_, which in turn acts as signaling molecules to activate defense response against pathogens. Secondly, the *SlSR1*- and *SlSR3L*-silenced plants constitutively expressed defense genes and PTI marker genes. Similar to the observations that the Arabidopsis *sr1* and rice *oscbt* mutant plants constitutively expressed a diverse set of defense genes [17,19,20], constitutive high levels of expression of defense genes including *SlPR1a*, *SlPR1b*, *SlPR-P2*, *SlPIN2* and *SlLapA*, which are regulated by different signaling pathways, were observed in the *SlSR1*- and *SlSR3L*-silenced plants (Figure 6B). In addition, the expression of the PTI marker genes including *Pti5*, *Lrr22* and *WRKY28* [34,35] was also significantly upregulated in the *SlSR1*- and *SlSR3L*-silenced plants (Figure 6C). These data indicate that both SlSR1 and SlSR3L negatively regulate PTI response. In this regarding, silencing of either *SlSR1* or *SlSR3L* would relieve their suppression on PTI response and thus lead to increased resistance against multiple pathogens including *B. cinerea* and *Pst* DC3000 (Figures 4 and 5). This is supported by general knowledge that PTI but not effector-triggered immunity (ETI) plays important roles in regulating immunity against necrotrophic fungal pathogens like *B. cinerea* [49] while both the PTI and ETI are require for immunity to biotrphic/hemibiotrophic pathogens such as *Pst* DC3000 [50]. Thirdly, the *SlSR1*- and *SlSR3L*-silenced plants constitutively activated the SA- and ET-mediated defense signaling pathways. It was previously found that the Arabidopsis *sr1* mutant plants contained high level of SA and had upregulated expression of defense and signaling genes [19,20] and that AtSR1 can bind to the CGCG box in the promoters of *EDS1*, *NDR1* and *EIN3* [20,21], indicating the involvements of AtSR1 in the SA- and ET-mediated signaling pathways. Our qRT-PCR analyses of expression of some defense signaling pathway-associated genes also demonstrated that both SlSR1 and SlSR3L have functions that negatively regulate the SA- and ET-mediated signaling pathways in tomato, as revealed by the upregulated expression of the *SlNPR1*, *SlEDS1*, *SlTGA1*, *SlETR4* and *SlERF1* genes in the *SlSR1*- and *SlSR3L*-silenced plants (Figure 6C). This is partially supported by the facts that the expression of *SlSR1* and *SlSR3L* in tomato fruits was induced rapidly by exogenously applied SA and ET [29,30]. In particular, it was shown that the Arabidopsis AtSR1 could bind to the CGCG box, a characteristic *cis*-elements for the SR proteins [12], in the promoter region of *EDS1* and suppressed the expression of *EDS1* [20], which is critical to biosynthesis of SA. Bioinformatics analysis also identified a CGCG box within 1.3 Kb from the starting codon in the promoter of tomato *SlEDS1* gene [30]. It is thus possible that SlSR1 and perhaps SlSR3Ll regulate negatively the expression of *SlEDS1* through binding to the CGCG box as the expression level of *SlEDS1* was upregulated when either *SlSR1* or *SlSR3L* was silenced (Figure 6C). Furthermore, the expression of *SlJAZ1*, known to be associated with the JA-mediated signaling pathway [39], was downregulated in the *SlSR1*- and *SlSR3L*-silenced plants (Figure 6C), suggesting a negative impact of SlSR1 and SlSR3L on the JA-mediated signaling pathway. This is in agreement with the observations that the Arabidopsis AtSR1 is a negative regulator for JA biosynthesis and herbivory tolerance [22,23]. Another possibility that the constitutively activated SA-mediated signaling pathway in the *SlSR1*- and *SlSR3L*-silenced plants antagonistically suppressed the JA-mediated signaling pathway cannot be ruled out because the antagonistic cross-talk between these two signaling pathways is a common phenomenon occurred in regulating defense response against infection by different pathogens [51]. Taken together, these data suggest that both SlSR1 and SlSR3L negatively regulate basic immunity in tomato through modulating the SA- and ET-mediated signaling pathways.

The involvement of the SR family members in abiotic stress was recently investigated using Arabidopsis knockout mutant lines [25,27]. Our VIGS-based functional analyses discovered that silencing of *SlSR1L* resulted in decreased drought stress tolerance (Figures 7E and 8A). SlSR1L is phylogenetically related to the Arabidopsis AtSR2 [29], which was shown to regulate drought stress responses [27]. Thus, it is likely that SlSR1L plays an important role in regulation of drought stress tolerance in tomato. The expression of *SlSR1L* was induced significantly by drought stress in detached leaves and in whole plants (Figure 7A and D). The decreased drought tolerance in the *SlSR1L*-silenced plants might be caused by multiple factors including morphological, physiological and molecular changes, which are affected by loss-of-function of *SlSR1L*. Like the stunted primary root in the Arabidopsis *sr2* mutant plants [27], the *SlSR1L*-silenced plants had limited root system and biomass under normal growth condition (Figure 8C and D). Although the exact function of *SlSR1L* in development of the root system in tomato needs to be further investigated, the limited root system in the *SlSR1L*-silenced plants may result in reduced capacity of water uptake. Another fact that affected the water status in the *SlSR1L*-silenced plants was the accelerated rate of water loss, as revealed in the detached leaves (Figure 8B). Furthermore, the expression of *SlAREB1* [42], *SlDREB* [43], *SlSpUSP* [44], *SlAREB2* [42] and *SGN-213276* [40] was suppressed in the *SlSR1L*-silenced plants (Figure 8E), indicating that SlSR1L may regulate the expression of a large set of drought stress-responsive genes. Recent microarray-based analyses of gene expression profiling between the Arabidopsis *sr1* and wild type plants revealed that hormone-mediated signaling such as ABA-mediated signaling plays important roles in AtSR1-regulated abiotic stress response [18,27]. However, exogenous ABA did not induce the expression of *SlSR1L* in tomato (Figure 7B) and *AtSR2* in Arabidopsis [12]. Thus, it is likely that, as a transcription factor, SlSR1L acts in a yet-unknown signaling pathway, in which some hormones such as ABA are involved, to regulate drought stress response in tomato.

Our data presented in this study clearly demonstrate that SlSR1/SlSR3L and SlSR1L play important roles in biotic and abiotic stress responses, respectively. However, several questions regarding the mechanism of action of SlSR1/SlSR3L and SlSR1L in biotic and abiotic stress response need to be addressed. Further identification of downstream target genes regulated by SlSR1/SlSR3L and SlSR1L will help to elucidate the molecular mechanisms and the signaling pathways involved in the SlSR1/SlSR3L-regulated defense response against pathogens and the SlSR1L-regulated drought stress response. Of particular, the SlSR3L will be a priority for further study because the function of its orthologs in other plants such as the Arabidopsis AtSR3 and AtSR6 [29] has not been defined yet. On the other hand, biochemical studies have shown that the Arabidopsis AtSR2/CAMTA1 [11], rice OsCBT [14] and tomato SlSR1 and SlSR3L (Figure 9) are functionally transcriptional activators in yeast. However, the Arabidopsis AtSR1 was shown to bind to the CGCG box in the promoters of the *EDS1*, *NDR1* and *EIN3* genes and repress their expression [20,21]. Thus, the biochemical mechanism regarding how the SR proteins as transcriptional activators repress the expression of the target genes after binding to the CGCG box in the promoters of these genes needs to be further investigated.

## Conclusion

Tomato genome encodes seven *SlSR* genes and expression of *SlSR1* and *SlSR3L* was significantly induced by *B. cinerea* and *Pst* DC3000. Silencing of either *SlSR1* or *SlSR3L* resulted in enhanced resistance to *B. cinerea* and *Pst* DC3000 and led to constitutive accumulation of H_2_O_2_, elevated expression of defense genes, PTI marker genes and regulatory genes involved in the SA- and ET-mediated signaling pathways. Meanwhile, expression of *SlSR1L* was significantly induced by drought stress and silencing of *SlSR1L* led to decreased drought stress tolerance. These results demonstrate that both SlSR1 and SlSR3L in the tomato *SlSR*/*CAMTA* family are negative regulators of defense response against *B. cinerea* and *Pst* DC3000 while SlSR1L is a positive regulator of drought stress tolerance in tomato.

## Methods

### Plant growth and treatments

Tomato (*Solanum lycopersicum*) cv. Suhong 2003 was used for all experiments. Seeds were scarified on moist filter paper in Petri dishes for 2 days and the sprouted seeds were transferred into a mixture of perlite: vermiculite: plant ash (1:6:2). All tomato plants were grown in a growth room at 22–24°C with 60% relative humidity (RH) under a 14 hr light (350 μmol · s^-1^ · m^-2^ photons m^-2^ sec^-1^)/10 hr dark cycle. Two-week-old plants were used for VIGS assays and four-week-old plants were used for other experiments. For analysis of gene expression in response to pathogen infection, whole plant inoculation assays for *B. cinerea* and vacuum infiltrated inoculation assays for *Pst* DC3000 along with corresponding mock-inoculation controls were performed (see below). For analysis of gene expression in drought stress, drought stress was applied to the plants by stopping watering for a period until wilting symptom appeared and normally watered plants were used as controls in the whole plant drought stress assays. Alternatively, fully expanded leaves were detached and subjected to drought stress treatment by placing on lab blench or on water-saturated filter papers in Petri dishes as controls in detached leaf assays. For ABA treatment, a solution of ABA (100 μM) or same volume of water as a control was sprayed onto leaf surface of the tomato plants. Leaf samples were collected at indicated time points after treatment or inoculation and stored at -80°C until use.

### Plant inoculation and disease assays

*Pst* DC3000 was grown overnight in King’s B (KB) liquid medium containing rifampicin at 50 μg/ml. The bacteria were collected and resuspended in 10 mM MgCl_2_ to OD_600_ = 0.0002 for plant inoculation. Four-week-old plants were vacuum infiltrated with suspension of *Pst* DC3000 or with MgCl_2_ solution as a mock inoculation control. The inoculated plants were kept in a sealed container to maintain high humidity (RH > 90%) and disease progress was observed daily. Leaf samples were collected from at least six *Pst* DC3000-inoculated or mock-inoculated plants at different time points after inoculation and used for analysis of gene expression and *in planta* bacterial growth. For measurement of bacterial growth, leaf discs (6 mm in diameter) were surface sterilized in 70% ethanol for 10 s, homogenized in 200 μl of 10 mM MgCl_2_, diluted in 10 mM MgCl_2_, and plated on KB agar plates containing rifampicin at 50 μg/ml. The plates were incubated at 28°C and the bacterial numbers were counted 3 days after incubation.

*B. cinerea* was grown on 2× V8 agar (36% V8 juice, 0.2% CaCO_3_, and 2% agar) at 22°C and spores were collected and resuspended in 1% maltose buffer to 2 × 10^5^ spores/ml for whole plant inoculation and 1 × 10^5^ spores/ml for detached leaf inoculation. Whole plant inoculation and detached leaf inoculation assays were performed according to previously reported procedure [52,53]. In the detached leaf inoculation assays, leaves were detached from at least 10 four-week-old VIGS-infiltrated plants and inoculated by dropping 5 μl of spore suspension on leaf surface. In the whole plant inoculation assays, four-week-old plants were inoculated by foliar spraying with spore suspension of *B. cinerea* or with same volume of 1% maltose buffer as a mock-inoculation control and leaf samples were collected at different time points after inoculation for analysis of gene expression and *in planta* fungal growth. The inoculated leaves and plants were kept at 22°C in sealed containers to retain the moist conditions favorable for disease development. *In planta* fungal growth was analyzed by the amplification of the transcripts of a *B. cinerea* Actin gene as a marker [53,54] using a pair of primers *BcActin*-F and *BcActin*-R (Additional file 2). Relative fungal growth was expressed as folds of the transcript levels of *BcActin* vs the transcript levels of a tomato *Actin* gene.

### Construction of vectors and VIGS assays

Based on the cDNA sequences of all seven *SlSR* genes [29], fragments of 386–462 bp for each *SlSR* gene (Additional file 3) were amplified with gene-specific primers (Additional file 2) from cDNAs synthesized from total RNA prepared from tomato leaf samples. After cloning and sequencing, these VIGS fragments were cloned into pTRV2 vector [32], yielding pTRV2-SlSRs. These pTRV2-SlSR constructs were then introduced into *Agrobacterium tumefaciens* strain GV3101 by electroporation using GENE PULSER II Electroporation System (Bio-Rad Laboratories, Hercules, CA, USA). Agrobacteria carrying pTRV2-GUS (as a negative control), pTRV2-PDS (as a positive control for silencing efficiency examination) or pTRV2-SlSRs were cultivated in YEP medium (10 g/l peptone, 10 g/l yeast extract, 5 g NaCl/l, 50 μg/ml rifampicin, 50 μg/ml kanamycin and 25 μg/ml gentamicin) for 36 hr with continuous shaking in a 28°C incubator. Cells were centrifuged and resuspended in infiltration buffer (10 mM MgCl_2_, 150 μM acetosyringone, MES, pH5.7). The agrobacteria carrying pTRV2-GUS, pTRV2-PDS or pTRV2-SlSR were mixed with the agrobacteria carrying pTRV1 in a ratio of 1:1 and maintained at OD_600_ = 1.5 for 3 hr at room temperature. The mixed agrobacteria suspension was infiltrated into the abaxial surface of the 2-week-old seedlings using 1 mL needleless syringes. Efficiency of the silencing procedure was evaluated based on the appearance of bleaching phenotype in the pTRV2-PDS-infiltrated plants [32]. When more than 90% of the pTRV2-PDS-infiltrated plants showed bleaching phenotype, the pTRV2-GUS- or pTRV2-SlSR-infiltrated plants in an independent experiment with same VIGS procedure were used for further study. Leaf samples were collected at 2 weeks after VIGS infiltration and used for analysis of the silencing efficiency by qRT-PCR.

### Subcellular localization

The coding sequences of *SlSR1* and *SlSR3L* were PCR amplified using pairs of gene-specific primers SlSR1-3F/SlSR1-3R and SlSR3L-3F/SlSR3L-3R, respectively (Additional file 2). After confirmation by sequencing, the coding regions of the *SlSR1* and *SlSR3L* genes were cloned into pFGC-Egfp. The recombinant plasmids pFGC-Egfp-SlSR1, pFGC-Egfp-SlSR3L and pFGC-Egfp were transformed into *A. tumefacies* strain GV3101 and the transformed agrobacteria were infiltrated individually into leaves of four-week-old *N. benthamiana* plants using 1-ml needless syringes. These agroinfiltrated plants were allowed to grow in a growth chamber at 25°C for 48 hr, and the GFP fluorescence was examined under a Leica TCS SP5 laser confocal microscope with excitation wavelength of 488 nm.

### Transcription activation assay in yeast

The coding sequences of *SlSR1* and *SlSR3L* were amplified using gene-specific primers (Additional file 2) and cloned into pBD-GAL4Cam vector to yield pBD-SlSR1 and pBD-SlSR3L. These two recombinant plasmids and the pBD empty vector (as a negative control) were transformed into yeast strain AH109. The transformed yeasts were confirmed by colony PCR and then cultivated on the SD/–Trp and SD/–Trp/–His medium for 3 days at 28°C, followed by addition of x-α-gal (5-bromo-4-chloro-3-indolyl-α-D-galactopyranoside). Transactivation activity of the fused proteins was evaluated according to the growth situation and production of blue pigments after the addition of x-α-gal of the transformed yeast cells on the SD/–Trp/–His medium.

### Detection of H_2_O_2_ accumulation

Detection of *in situ* H_2_O_2_ was carried out using DAB staining method as described previously [55]. Leaves were collected from pTRV2-SlSR1- or pTRV2-SlSR3L-infiltrated and pTRV2-GUS-infiltrated plants and dipped into DAB solution (1 mg/ml, pH3.8) and incubated for 8 hr in dark at room temperature. Thereafter, leaves were placed into acetic acid/glycerol/ethanol (1:1:1, vol/vol/vol) and boiled for 5 min in a water bath. After several changes of the solution, then leaves were maintained in 60% glycerol and accumulation of H_2_O_2_ in leaves was photographed using a digital camera.

### Drought tolerance assays

At least 10 individual pTRV2-SlSRs- or pTRV2-GUS-infiltrated plants were used in each experiment. Drought stress was applied to the tomato plants by stopping watering for a certain period of 10–12 days or until the wilting symptoms were obvious. To assess the ratio of water loss, fully expanded leaves from 6 individual pTRV2-SlSR1L- or pTRV2-GUS-infiltrated plants were detached and placed on the bench top. The weights of the leaves were recorded at different time points after detachment and the average water loss ratio was calculated by comparing with the initial weights. Roots from 6 individual pTRV2-SlSR1L- or pTRV2-GUS-infiltrated plants were cut, cleaned and dried in 70°C oven for 24 hr and the final weight was calculated and compared. Leaf samples were collected from the pTRV2-SlSRs- or pTRV2-GUS-infiltrated plants at 0 hr (as unstressed control) and 7 days after stopping watering and were subjected to analysis of gene expression.

### qRT-PCR analysis of gene expression

Total RNA was extracted from frozen leaf samples using TRIzol reagent (Invitrogen, Shanghai, China) and treated with RNase-free DNase (TaKaRa, Dalian, China) to erase any genomic DNA in the RNA samples. First-strand cDNA was synthesized from 0.6 μg total RNA using AMV reverse transcriptase (TaKaRa, Dalian, China) according to the manufacturer’s recommendations. Each qRT-PCR reaction contained 12.5 μl SYBR Premix Ex Taq (TaKaRa, Dalian, China), 0.1 μg cDNA and 7.5 pmol of each gene-specific primer (Additional file 2) in a final volume of 25 μl, and run on three independent biological replicates. The qRT-PCR was performed in a CFX96 real-time PCR detection system (BioRad, Hercules, CA, USA) and relative expression levels were calculated using the 2^-ΔΔCT^ method. The expression level of a tomato actin gene was used as an internal control to normalize the expression data for the target genes. Relative expression levels of the target genes were shown as folds of the expression level of the actin gene or as folds of the expression levels in treated plants/control plants.

### Statistical analysis

All experiments were repeated independently three times and data were collected from experiments on three biological samples. All data obtained were subjected to statistical analysis according to the Student’s *t*-test and the probability values of *p* < 0.05 were considered as significant difference.

### Accession numbers for *SlSRs*

The tomato *SlSR* gene sequences used in this study were retrieved from GenBank under the following accession numbers: *SlSR1*, GU170838; *SlSR1L*, JN558810; *SlSR2*, JN566047; *SlSR2L*, JN566048; *SlSR3*, JN566049; *SlSR3L*, JN566051; *SlSR4*, JN566050. These *SlSR* gene sequences were deposited by Yang et al. [29].

### Availability of supporting data

The sequences of SlSR proteins and sequences of VIGS fragments for *SlSR* genes used in this study are included in Additional files 1 and 3.

## Additional files

Additional file 1:
**Alignment of tomato SlSR protein sequences and positions of the VIGS fragments in the SlSR proteins.** The GenBank accession numbers for SlSR proteins are as follows: SlSR1, ADK47999; SlSR1L, AEX31181; SlSR2, AEX07774; SlSR2L, AEX07775; SlSR3, AEX07776; SlSR3L, AEX07778; SlSR4, AEX07777. These SlSR protein sequences were deposited by Yang et al. [29]. Regions selected for the VIGS fragments of each of SlSR genes were underlined with red lines. Additional file 2: Table S1. Primers used in this study. Additional file 3:
**Sequences of the VIGS fragment for** ***SlSR*** **genes.**
