# Supplementary material for: Tomato SR/CAMTA transcription factors SlSR1 and SlSR3L negatively regulate disease resistance response and SlSR1L positively modulates drought stress tolerance
Source: BMC Plant Biol. 2014 Oct 28;14:286. doi: 10.1186/s12870-014-0286-3 (PMC4219024; doi:10.1186/s12870-014-0286-3)

**Additional file 1 Alignment of tomato SlSR protein sequences and positions of the VIGS fragments in the SlSR proteins.**

The corresponding GenBank protein accession numbers for SlSR proteins are as follows: SlSR1, ADK47999; SlSR1L, AEX31181; SlSR2, AEX07774; SlSR2L, AEX07775; SlSR3, AEX07776; SlSR3L, AEX07778; SlSR4, AEX07777. These SlSR protein sequences were deposited by Yang et al. [29]. Regions selected for the VIGS fragments of each of SlSR genes were underlined with red lines.


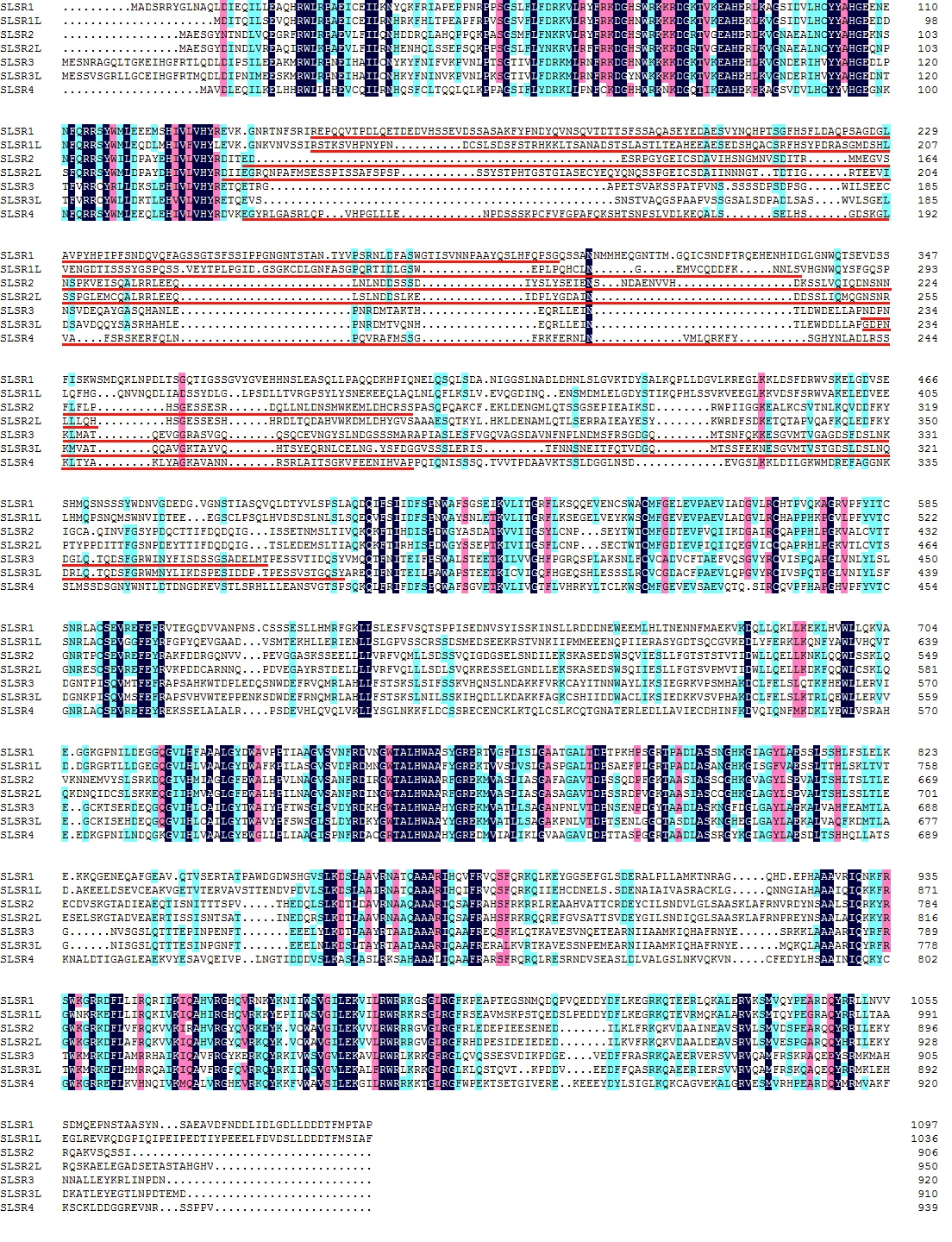

Supplement: Additional file 1: — Alignment of tomato SlSR protein sequences and positions of the VIGS fragments in the SlSR proteins. The GenBank accession numbers for SlSR proteins are as follows: SlSR1, ADK47999; SlSR1L, AEX31181; SlSR2, AEX07774; SlSR2L, AEX07775; SlSR3, AEX07776; SlSR3L, AEX07778; SlSR4, AEX07777. These SlSR protein sequences were deposited by Yang et al. [29]. Regions selected for the VIGS fragments of each of SlSR genes were underlined with red lines. [file 12870_2014_286_MOESM1_ESM.doc]
