# Supplementary material for: Tomato SR/CAMTA transcription factors SlSR1 and SlSR3L negatively regulate disease resistance response and SlSR1L positively modulates drought stress tolerance
Source: BMC Plant Biol. 2014 Oct 28;14:286. doi: 10.1186/s12870-014-0286-3 (PMC4219024; doi:10.1186/s12870-014-0286-3)
Supplement: Additional file 2: Table S1. — Primers used in this study. [file 12870_2014_286_MOESM2_ESM.doc]

**Additional file 2 Table S1 Primers used in this study.**

| Primers | Sequences (5’-3’) | Size (bp) |
| --- | --- | --- |
| *Cloning of cDNA* | | |
| SlSR1-F | ATGGCAGACAGTAGGCGTTA | 3294 |
| SlSR1-R | TCAAGGTGCTGTAGGCATAAAA |
| SlSR1L-F | ATGGACATAACACAGATATTATCCG | 3114 |
| SlSR1L-R | TTATTCAAATGCTATAGACATGAAAGTA |
| SlSR2-F | ATGGCAGAATCAGGATACAACACA | 2721 |
| SlSR2-R | TTAGACATGTCCATGAGCAGTTG |
| SlSR2L-F | ATGGCGGAATCAGGATATGATATT | 2853 |
| SlSR2L-R | CTAGATGGATGATTGACTGACCT |
| SlSR3--F | ATGGAAAGCAACAGAGCAGGAC | 2763 |
| SlSR3-R | CTAGTTATCAGGATTGATAAGCCTT |
| SlSR3L-F | ATGGAAAGTAGCGTATCAGGACGA | 2733 |
| SlSR3L-R | TTAGTCCATCTCAGTGTCAGGATTG |
| SlSR4-F | ATGGCAGTAGATCTTGAACAGATA | 2820 |
| SlSR4-R | CTAAACTGGTGGTGATGACCTA |
| *VIGS constructs* | | |
| SlSR1-VIGS-F | TGCTCTAGA GAACCTCAACAAGTTACTCC | 462 |
| SlSR1-VIGS-R | CCGGAGCTC GCCAGAAGGCTGAAAATGGA |
| SlSR1L-VIGS-F | TGCTCTAGA AGCACCAAATCAGTGCACCC | 438 |
| SlSR1L-VIGS-R | CCGGAGCTC TGACAAATTGTTCTTGAAAT |
| SlSR2-VIGS-F | TGCTCTAGA ATGAATCTAGACCTGGATAT | 389 |
| SlSR2-VIGS-R | CCGGAGCTC CGGAGAGCTCCTGCAGTGAT |
| SlSR2L-VIGS-F | TGCTCTAGA CATAATCGAGGGTAGGCAGA | 400 |
| SlSR2L-VIGS-R | CCGGAGCTC ATGTTGCAACAGCAGTCTAT |
| SlSR3-VIGS-F | TGCTCTAGA ATCCTAACAAGCTAATGGCA | 386 |
| SlSR3-VIGS-R | CCGGAGCTC AGTCATCAGCTCATCTGCAG |
| SlSR3L-VIGS-F | TGCTCTAGA ATCCCAACAAGATGGT TGCT | 386 |
| SlSR3L-VIGS-R | CCGGAGCTC ATATGATTGACCTGTTGACA |
| SlSR4-VIGS-F | TGCTCTAGA ACAGACTAGGTGCCTCCCGT | 455 |
| SlSR4-VIGS-R | CCGGAGCTC AGGAGCAACATGGATATTCT |
| *Transient expression* | | |
| SlSR1-GFP-F | AGT GGATCCATGGCAGACAGTAGGCGTTA | 3294 |
| SlSR1-GFP-R | GCG TCTAGATCAAGGTGCTGTAGGCATAA |
| SlSR3L-GFP-F | AGT GGATCCATGGAAAGTAGCGTATCAGG | 2733 |
| SlSR3L-GFP-R | GCG TCTAGATTAGTCCATCTCAGTGTCAG |
| *qRT-PCR* | | |
| SlSR1-RT-F | CGGGAAGTAAAGGGTAACAG | 128 |
| SlSR1-RT-R | AATTTTGCAGACGCGGAACT |
| SlSR1L-RT-F | CTATTTGGAAGTCAAGGGTA | 100 |
| SlSR1L-RT-R | ACTATCGGATAATGAGCAGT |
| SlSR2-RT-F | TCCTGCCTACGAGCACATTG | 122 |
| SlSR2-RT-R | CCGAGACATTCATCCCATTA |
| SlSR2L-RT-F | TTGAAAGAAATTGATCCCCTCT | 171 |
| SlSR2L-RT-R | ATCCAGCATATCCTTCCACA |
| SlSR3-RT-F | AGATGAACAAGCGTATGGTG | 182 |
| SlSR3-RT-R | CAACTGAAGCTCTTCCTCCC |
| SlSR3L-RT-F | CACATTTAGAACCTAACAGGGATA | 187 |
| SlSR3L-RT-R | TCACATAGATTGCGTTGCTC |
| SlSR4-RT-F | TTCAGAGGCGAAGTTATTGG | 154 |
| SlSR4-RT-R | TTGGAACTACTGTCGGGATT |
| SlActin-RT-F | CCAGGTATTGCTGATAGAATGAG | 113 |
| SlActin-RT-R | GAGCCTCCAATCCAGACAC |
| SlPIN2-RT-F | CATCTTCTGGATTGCCCA | 106 |
| SlPIN2-RT-R | ACACACAACTTGATGCCCAC |
| SlLapA-RT-F | GGGACTAATGATGTTTGGAA | 109 |
| SlLapA-RT-R | GTGGCAATTTTATTTAGGCA |
| SlPR1b-RT-F | TTTCCCTTTTGATGTTGCT | 96 |
| SlPR1b-RT-R | TGGAAACAAGAAGATGCAGT |
| SlPR-P2-RT-F | CGATCTAAATTGATTTCATAGTACG | 116 |
| SlPR-P2-RT-R | TCGTGAAGGATATACAAAATACA |
| BcActin-RT-F | CGTCACTACCTTCAACTCCATC | 107 |
| BcActin-RT-R | CGGAGATACCTGGGTACATAGT |
| SGN-21477-RT-F | GGTGTTTGTGCTGACCTACT | 100 |
| SGN-21477-RT-R | CTTCCAAATCAGCCAAACCTTC |
| SGN-213276-RT-F | GTCAAACACTGGAAAGCATGAA | 110 |
| SGN-213276-RT-R | AGCTGCTCCACTTGTCTTATC |
| SlAREB1-RT-F | GTGGTGGGAAGGATGGAAATA | 120 |
| SlAREB1-RT-R | CTCTCACAACTCCAGCTCTAAC |
| SlAREB2-RT-F | CATGTGGTGAAGGTGGAAGA | 98 |
| SlAREB2-RT-R | CGCAGACTCCCTGTTCTTTAT |
| SlDREB-RT-F | CGGAGGAACTGGGTGAAATTA | 99 |
| SlDREB-RT-R | CGTCCACTGAATCACTGATCTT |
| SpUSP-RT-F | CGCGGCAAGAGAGAATACAT | 95 |
| SpUSP-RT-R | CTCTCATCGATAGCCACCATTATC |
| SlGRX1-RT-F | GTACGTCAGCCTCCAAAGAA | 103 |
| SlGRX1-RT-R | CCTTCACTAGCCGGTCAATAA |
| SlWfi-RT-F | AGGGAATGATAGAGCGTCG | 143 |
| SlWfi-RT-R | CATCGTCATTGGACTTGGC |
| SlRboh1-RT-F | TGAGGAAGAGAAGCCCAATAAG | 91 |
| SlRboh1-RT-R | CACAAGACCAGAACCCAAATTC |
| SlCAT-RT-F | CCC AGT TAA TGC TCC CAA GTG T | 118 |
| SlCAT-RT-R | AGG ACG ACA AGG ATC AAA CCT C |
| SlSOD-RT-F | GGC CAA TCT TTG ACC CTT TAT G | 183 |
| SlSOD-RT-R | AAG TCC AGG AGC AAG TCC AGT T |
| SlAPX-RT-F | ACT TCA CGG AGC TTT TGA GTG G | 141 |
| SlAPX-RT-R | CAG CAT AGT CAG CAA AGA AGG C |
| SlPR1a-RT-F | GGC AGG AAC ACC AAA GAA ACC A | 127 |
| SlPR1a-RT-R | TGG CCT CTG GTC AGG TTT AAA G |
| SlNPR1-RT-F | TGT GGG AAA GAT AGC AGC ACG | 147 |
| SlNPR1-RT-R | GTC CAC ACA AAC ACA CAC ATC |
| SlJAZ7-RT-F | TAG CCA CAC TGG AGG CAA GAT T | 140 |
| SlJAZ7-RT-R | CTA GGT CTA ATT CCA TGA GCG C |
| SlETR4-RT-F | CTG AAG ATG GGC AAA GGA TG | 127 |
| SlETR4-RT-R | ACA CGA GGT TGT TGA TGA GG |
| SlTGA1-RT-F | GGCATGTGGGATGATTTC | 116 |
| SlTGA1-RT-R | CATCATCGGTATCTGGTCCT |
| SlERF1-RT-F | TGG AGT TAG AAA GAG GCC ATG G | 143 |
| SlERF1-RT-R | CCC TCA TTG ATA ATG CGG CTT G |
| SlEDS1-RT-F | GAATGACCTTGGCCTGAGTACAAG | 114 |
| SlEDS1-RT-R | CCTGCTGCACGAAGACACAG |
| SlLrr22-RT-F | AAGATTGGAGGTTGCCATTGGAGC | 100 |
| SlLrr22-RT-R | ATCGCGATGAATGATCGGTGGAGT |
| SlPti5-RT-F | ATTCGCGATTCGGCTAGACATGGT | 95 |
| SlPti5-RT-R | AGTAGTGCCTTAGCACCTCGCATT |
| SlWRKY28-RT-F | ACAGATGCAGCTACCTCATCCTCA | 125 |
| SlWRKY28-RT-R | GTGCTCAAAGCCTCATGGTTCTTG |
| *Transcription activation assay in yeast* | | |
| SlSR1-Yeast-F | AGT GAATTCATGGCAGACAGTAGGCGTTA | 3294 |
| SlSR1-Yeast-R | GCG CTGCAGTCAAGGTGCTGTAGGCATAA |
| SlSR3L-Yeast-F | AGT GAATTCATGGAAAGTAGCGTATCAGG | 2733 |
| SlSR3L-Yeast-R | GCG CTGCAGTTAGTCCATCTCAGTGTCAG |
