# Supplementary material for: Tomato SR/CAMTA transcription factors SlSR1 and SlSR3L negatively regulate disease resistance response and SlSR1L positively modulates drought stress tolerance
Source: BMC Plant Biol. 2014 Oct 28;14:286. doi: 10.1186/s12870-014-0286-3 (PMC4219024; doi:10.1186/s12870-014-0286-3)
Supplement: Additional file 3: — Sequences of the VIGS fragment for SlSR genes. [file 12870_2014_286_MOESM3_ESM.doc]

**Additional file 3 Sequences of the VIGS fragment for *SlSR* genes.**

SlSR1-vigs: 462 bp

GAACCTCAACAAGTTACTCCTGATTTGCAAGAAACTGATGAAGATGTACACAGCTCTGAGGTGGACAGTTCCGCGTCTGCAAAATTTTATCCAAATGATTACCAAGTGAACTCACAAGTCACTGACACAACTAGCTTCAGCAGTGCGCAGGCCTCAGAATATGAGGATGCCGAATCAGTGTACAATCAACATCCAACTTCTGGATTTCACTCATTCCTTGATGCTCAGCCAAGTGCTGGAGATGGACTTGCTGTACCTTATCATCCGATTCCCTTCTCAAATGATCAAGTACAGTTTGCAGGAAGTTCTGGTACGAGTTTCTCCTCAATCCCACCAGGAAATGGAAACACAAGCACAGCAAATACCTATGTACCCAGTAGGAACCTCGACTTCGCATCATGGGGGACCATTTCAGTTAATAATCCCGCTGCATATCAATCTCTCCATTTTCAGCCTTCTGGC

SlSR1L-vigs: 438 bp

AGCACCAAATCAGTGCACCCAAACTATCCGAATGACTGCTCATTATCCGATAGTTTTTCTACGAGACACAAGAAACTAACTTCAGCAAATGCTGATTCAACAAGTCTAGCAAGCACTTTAACAGAAGCACATGAAGAAGCTGAATCAGAAGATAGTCACCAAGCGTGTTCTAGATTTCATTCATATCCAGACCGAGCATCTGGAATGGACAGTCATCTAGTGGAGAATGGGGATACCATCAGTAGTTCATATGGTTCACCTCAATCTTCAGTGGAGTATACACCACTTCCTGGTATAGATGGATCGGGAAAGTGTGATCTTGGTAATTTTGCATCTGGTCCTCAAAGAACAATTGATTTGGGATCTTGGGAACCACTTCCTCAGCATTGTTTAAATGGTGAGATGGTATGCCAAGATGATTTCAAGAACAATTTGTCA

SlSR2-vigs: 389 bp

ATGAATCTAGACCTGGATATGGAGAAATTTGTTCTGATGCAGTTATCCATAGTAATGGGATGAATGTCTCGGACATCACCAGGATGATGGAGGGGGTGAGCAACTCACCGAAGGTTGAGATAAGTCAAGCATTGCGAAGACTTGAGGAGCAGTTAAATTTAAATGATGACAGCTCGTCGGATATTTATTCACTCTATAGTGAGATTGAGAACTCAAATGATGCTGAAAATGTCGTGCATGACAAAAGTTCACTTGTCCAGATCCAGGACAATTCAAATAATTTTCTGTTCCTGCCTCATTCAGGTGAGAGCAGTGAATCTCGGGATCAGCTTTTGAACCTGGATAATAGCATGTGGAAAGAGATGCTGGATCACTGCAGGAGCTCTCCG

SlSR2L-vigs: 400 bp

CATAATCGAGGGTAGGCAGAATCCAGCCTTCATGTCAGAATCTTCTCCAATTTCTTCTGCTTTCTCTCCTAGTCCAAGCTCCTATTCTACTCCACATACGGGCTCTACTGGCATTGCAAGTGAATGTTATGAGCAATACCAGAACCAATCTAGCCCTGGAGAAATTTGCTCTGATGCAATCATCAATAATAACGGGACGACAGACACCATAGGGAGAACAGAGGAGGTCATCAGTTCTCCGGGGCTTGAGATGTGTCAAGCATTGCGAAGGCTCGAGGAGCAGTTAAGTTTAAATGATGACAGCTTGAAAGAAATTGATCCCCTCTATGGTGATGCAATTAATGACGACAGCTCACTTATCCAGATGCAGGGGAATTCAAATAGACTGCTGTTGCAACAT

SlSR3-vigs: 386 bp

ATCCTAACAAGCTAATGGCAACTCAAGAAGTGGGAGGAAGAGCTTCAGTTGGGCAACAGAGTCAATGTGAAGTTAATGGTTACAGCCTTAATGATGGCTCTTCATCCATGGCGAGAGCACCTATAGCATCCTTAGAAAGTTTTGTTGGCCAAGTGGCTGGCAGCGATGCTGTGAATTTTAATCCTTTAAATGACATGTCCTTCCGTTCGGGGGATGGTCAGATGACTTCAAACTTTCAGAAGAAAGAATCTGGAGTTATGACAGTGGGTGCAGGTGATTCTTTTGATAGTCTTAACAAGGATGGTCTCCAAACTCAAGACAGTTTTGGACGGTGGATCAACTACTTCATCAGTGATTCTTCGGGATCTGCAGATGAGCTGATGACT

SlSR3L-vigs: 386 bp

ATCCCAACAAGATGGTTGCTACCCAGCAAGCAGTAGGTAAAACTGCATATGTACAGCACACGTCATATGAGCAACGCAATCTATGTGAATTAAATGGTTACAGCTTCGATGGTGGTGTCTCCTCTTCTCTTGAGAGAATTTCTACATTCAACAATTCAAATGAGATCACCTTCCAGACAGTGGATGGTCAAATGACTTCAAGTTTTGAGAAGAATGAGTCCGGAGTAATGACAGTGAGCACAGGCGATTCCTTGGATAGTCTGAACCAGGATAGACTTCAAACTCAGGATAGCTTTGGAAGGTGGATGAACTACCTTATCAAGGATTCTCCAGAATCCATAGACGATCCAACTCCCGAATCTTCAGTGTCAACAGGTCAATCATAT

SlSR4-vigs: 455 bp

ACAGACTAGGTGCCTCCCGTCTGCAACCTGTACATCCAGGGTTATTGCTTGAAAATCCCGACAGTAGTTCCAAGCCTTGCTTTGTATTTGGCCCAGCATTTCAGAAATCTCATACATCAAATCCAAGCTTAGTTGACTTGAAAGAACAAGCACTCTCTTCTGAACTTCACAGTGGTGATTCCAAAGGACTTGTGGCGTTTTCGAGGTCAAAGGAGAGGTTTCAGCTCAATCCACAAGTTAGAGCATTCATGTCTTCTGGCTTTCGTAAATTTGAAAGAAATTTAAATGTTATGCTACAGAGAAAGTTCTACTCGGGACACTATAATCTGGCTGATTTGCGGTCCAGTAAACTCACTTATGCAAAATTATATGCTGGTAAAGCTGTGGCGAACAACAGAAGCAGATTGGCAATTACTTCTGGAAAAGTATTTGAGGAGAATATCCATGTTGCTCCT
